# Supplementary material for: WordCommentsAnalyzer: A windows software tool for qualitative research
Source: F1000Res. 2018 Sep 4;7:536. Originally published 2018 May 3. [Version 2] doi: 10.12688/f1000research.14819.2 (PMC6137411; doi:10.12688/f1000research.14819.2)
Supplement: Supplementary file 3 [file f1000research-7-17614-s0002.tgz › b2a79496-4ada-47c4-9682-a99ce867836d.zip!/Eight Iranian Health Journals/Journal of Research in Rehabilitation Sciences _34.docx]

© Journal of Research in Rehabilitation Sciences . This work is licensed under CC BY-NC 4.0 (https://creativecommons.org/licenses/by-nc/4.0/).

Volume & Issue: دوره 8، شماره 7: 1391 ویژه نامه گفتاردرمانی

Title: بررسی ارتباط بین افکار و باورهای نامناسب افراد دارای لکنت بزرگسال با میزان اضطراب و افسردگی آن‌ها

http://jrrs.mui.ac.ir/index.php/jrrs/article/view/868

مقدمه: لکنت اختلالی است که در جریان گفتار ایجاد می‌شود و تأثیرات شگرفی بر توانایی برقراری ارتباط فرد بر جای می‌گذارد. افکار و باورهای نامناسب در مورد لکنت به جنبه شناختی اضطراب مربوط است و وجود چنین باورهایی می‌تواند عامل ایجاد و یا تشدید اضطراب اجتماعی در افراد دارای لکنت باشد. شناسایی زودهنگام و درمان این افکار و باورها می‌تواند از شدت اضطراب اجتماعی فرد بکاهد. هدف از مطالعه حاضر، شناسایی فراوانی بروز افکار و باورهای نادرست در مورد لکنت و بررسی ارتباط این افکار با میزان اضطراب و افسردگی بود. مواد و روش‌ها: نمونه‌های این مطالعه شامل 52 فرد بزرگسال دارای لکنت بود. کلیه افراد پرسش‌نامه‌های UTBAS (Unhelpful thoughts and beliefs about stuttering)، BAI (Beck anxiety inventory) و BDI-II (Beck depression inventory) را تکمیل کردند و نتایج با استفاده از آماره‌های توصیفی و همبستگی رتبه‌ای Spearman مورد تجزیه و تحلیل قرار گرفت. یافته‌ها: افکار و باورهای نامناسب (از جمله احساس اجبار درونی برای کنترل لکنت، ترس از احتمال بروز لکنت در هر لحظه و همچنین تصور دقت دیگران به لکنت) به میزان متفاوت در کلیه افراد دارای لکنت وجود داشت. این افکار همبستگی مثبت و معنی‌داری با اضطراب (01/0 > P، 55/0 = r) و افسردگی (01/0 > P و 46/0 = r) داشتند. نتیجه‌گیری: بین باورها و افکار نادرست بررسی شده و نشانه‌های اضطراب و افسردگی ارتباط وجود دارد و همچنین همبستگی مثبت بین این عوامل حاکی از ضرورت توجه ویژه به شناسایی زودهنگام این افکار و درمان به موقع مسایل سلامت روانی در افراد دارای لکنت است.

Title: مقایسه رضايت‌مندی مراجعین از کلینیک‌های دولتی و خصوصی گفتار درمانی شهر اهواز

http://jrrs.mui.ac.ir/index.php/jrrs/article/view/508

مقدمه: در نظام توان‌بخشی (به ویژه گفتار درمانی) دستیابی به اهداف نهایی درمان، طولانی مدت است. بنابراین دانستن سطح رضایت‌مندی مراجعین در حفظ و ادامه مداخلات درمانی اهمیت دارد. هدف از این تحقیق، سنجش و مقایسه رضایت مراجعین به کلینیک‌های گفتار درمانی دولتی و خصوصی شهر اهواز در سال 1390 بود. مواد و روش‌ها: در مطالعه مقطعی مقایسه‌ای حاضر، 425 نفر از مراجعینی که حداقل 5 بار به یکی از کلینیک‌های گفتار درمانی دولتی یا خصوصی اهواز مراجعه کرده بودند، به تصادف انتخاب شدند. اطلاعات از طریق پرسش‌نامه ساختارمند طی مصاحبه حضوری یا تلفنی جمع‌آوری و نمره رضایت‌مندی هر فرد بر مبنای یک مقیاس 100-0 محاسبه گردید. تجزیه و تحلیل داده‌ها از طریق آزمون‌های آماری در نرم‌افزار SPSS نسخه 15 انجام شد. یافته‌ها: میانگین نمره رضایت کلی مراجعین کلینیک‌های خصوصی (91/4 ± 4/71) از کلینیک‌های دولتی (16/5 ± 6/69) بیشتر و تفاوت آن معنی‌دار بود (038/0 = P). مراجعین مراکز خصوصی از وضعیت فیزیکی کلینیک (001/0 = P)، کیفیت خدمات ارایه شده (018/0 = P) و نحوه برخورد پرسنل (008/0 = P) بیش از مشتریان مراکز دولتی رضایت داشتند. در مقابل مشتریان مراکز دولتی از دسترسی مکانی- زمانی (01/0 = P) و رضایت مالی (015/0 = P) رضایت بیشتری داشتند. نتیجه‌گیری: كلينيك‌هاي خصوصي مورد مطالعه نسبت به كلينيك‌هاي دولتي از كيفيت ارایه خدمات بالاتر، روابط اجتماعي بهتر و فضاي فيزيكي مناسب‌تري برخوردار هستند كه اين نتايج مي‌تواند به منظور ارتقای كمي و كيفي كلينيك‌هاي دولتي به كار گرفته شود.

Title: مقایسه ویژگی‌‌های آکوستیکی صوت در معلمان زن با و بدون شکایت صوتی در مدارس ابتدایی شهر تهران

http://jrrs.mui.ac.ir/index.php/jrrs/article/view/753

مقدمه: معلمان بزرگ‌ترین گروه کاربران حرفه‌ای صوت محسوب می‌شوند که به واسطه شرایط شغلی خاصی که دارند به میزان بیشتری در معرض ابتلا به اختلالات صوت قرار دارند. مطالعه حاضر با هدف تعیین و مقایسه ویژگی‌‌های آکوستیکی صوت در معلمان زن دارای شکایت صوتی و بدون شکایت صوتی صورت گرفت. مواد و روش‌ها: روش انجام این پژوهش توصیفی- تحلیلی، غیر مداخله‌ای و از نوع مقطعی- مقایسه‌ای است که بر روی 80 معلم زن مدارس ابتدایی آموزش و پرورش شهر تهران در دو گروه با و بدون شکایت صوتی صورت گرفت. اطلاعات زمینه‌ای از طریق مصاحبه استخراج شد و سپس ارزیابی آکوستیکی صوت از طریق کشش واکه /a/ صورت گرفت. میانگین فرکانس پایه، آشفتگی فرکانس، نسبت هارمونیک به نویز و آشفتگی شدت با استفاده از نرم‌افزار Praat نسخه 5.3.13 محاسبه شد و داده‌های به دست آمده از دو گروه مورد مقایسه آماری قرار گرفت. یافته‌ها: در تمامی‌متغیر‌‌های مورد بررسی که شامل میانگین، فرکانس پایه، آشفتگی فرکانس، نسبت هارمونیک به نویز و آشفتگی شدت بودند، تفاوت معنی‌داری بین دو گروه یافت نشد (05/0 < P). نتیجه‌گیری: نتایج این مطالعه نشان داد که الزاماً بین نتایج ارزیابی آکوستیکی صوت در دو گروه با و بدون شکایت صوتی تفاوت معنی‌داری وجود ندارد. به عبارت دیگر، شاید ویژگی‌‌های آکوستیکی صوت خود به تنهایی نمی‌توانند بین افراد دارای شکایت صوتی و بدون شکایت صوتی تمیز بگذارند و نیاز به استفاده از روش‌‌ها و شاخص‌‌های آکوستیکی کامل‌تری مانند شاخص شدت بدآوایی (Dysphonia severity index یا DSI) می‌باشد که خود مستلزم پژوهش‌‌های بیشتری است.

Title: بررسی رابطه بین نمرات شاخص کیفیت زندگی مرتبط با معلولیت صدا (VHI) و پارامترهای آکوستیکی در بیماران مبتلا به اختلالات صوتی متوسط تا شدید

http://jrrs.mui.ac.ir/index.php/jrrs/article/view/779

مقدمه: آسیب‌های صوتی می‌توانند ناشی از ناهنجاری‌های روانی، فیزیولوژیکی، آناتومیکی و عملکردی باشند که منجر به معلولیت در سایر جنبه‌های زندگی فرد می‌شوند. با توجه به این‌که شخصیت افراد، شغل آن‌ها و درک آن‌ها از اختلال، تأثیرات متفاوتی بر کیفیت زندگی آن‌ها دارد، در این مطالعه به بررسی رابطه بین علایم صوتی بیماران و تأثیر آن برکیفیت صوتی زندگی فرد پرداخته شد. مواد و روش‌ها: شرکت کنندگان در مطالعه حاضر، 40 نفر از بیماران مبتلا به اختلال صوتی متوسط تا شدید مراجعه کننده به کلینیک‌های گفتار درمانی بودند. نمونه صوتی شامل کشیدن واکه /a/ به مدت 10 ثانیه از بیمار گرفته شد و فرم VHI (Voice handicap index) به بیمار ارایه و از وی خواسته شد فرم را تکمیل نماید. سپس نمونه ضبط شده توسط برنامه Voice analysis از دستگاه Dr.Speech تجزیه و تحلیل شد و مقادیر  پارامترهای آکوستیکی صدا محاسبه و در نهایت میانگین این نمرات و نمرات آزمون VHI از طریق برنامه SPSS نسخه 16 وارد کامپیوتر گردید و با استفاده از آزمون ضریب همبستگی Pearson مورد تجزیه و تحلیل آماری قرار گرفت. یافته‌ها: بین پارامترهاي آکوستیکی نسبت سیگنال به نویز با نمره كلي ازمون (005/0 = P)، زيرآزمون عاطفي (008/0 = P)، زيرآزمون فيزيكي (014/0 = P) و زيرآزمون عملكردي (007/0 = P)، نسبت هارمونی به نویز با  نمره كلي آزمون (006/0 = P)، زيرآزمون عاطفي (009/0 = P)، زير آزمون فيزيكي (014/0 = P) و زيرآزمون عملكردي (008/0 = P)، پارامتر آکوستیکی انحراف معیار فرکانس پایه با نمره كلي آزمون (041/0 = P)،  زيرآزمون فيزيكي (013/0 = P) و زيرآزمون عملكردي (044/0 = P) و آشفتگي دامنه با نمره كلي آزمون (031/0 = P) و زيرآزمون فيزيكي (009/0 = P) ارتباط معنی‌داری وجود داشت. نتیجه‌گیری: در این مطالعه بین نمرات VHI و مقادیر آکوستیکی اندازه‌گیری شده در جامعه مورد مطالعه یا ارتباطی مشاهده نشده یا ارتباط دیده شده ضعیف بود. دلایل این ارتباط ضعیف می‌تواند به عواملی برگردد که درک فرد از معلولیت را تحت تأثیر قرار می‌دهد و شاید شامل شخصیت بیمار، سیر پیشرفت بیماری، تجربیات گذشته عملکردهای صوتی، شغل، وضعیت اجتماعی و ... ‌باشد.

Title: بررسی مقدماتی تأثیر مداخله زودهنگام روی گفتار کودکان دارای شکاف کام

http://jrrs.mui.ac.ir/index.php/jrrs/article/view/754

مقدمه: کودکان دارای شکاف کام خزانه محدودی از همخوان‌ها دارند و خطاهای جبرانی یک نگرانی ویژه در آن‌ها می‌باشد که این امر روی رشد اولیه زبانشان تأثیر می‌گذارد. گفتار درمانی اغلب بعد از سه سالگی برای رفع این خطاها شروع می‌شود و بسیار زمان‌بر و پرهزینه است. تاکنون در داخل کشور مطالعه‌ای در مورد پیشگیری از ایجاد این خطاها و یا رفع آن‌ها قبل از تثبیتشان انجام نشده است. هدف از این مطالعه، بررسی تأثیر مداخله زودهنگام با استفاده از روش تحریک متمرکز روی گفتار کودکان دارای شکاف کام بود. مواد و روش‌ها: مطالعه حاضر به شیوه تک موردی، روی 4 کودک دارای شکاف کام انجام شد. ابتدا از این کودکان نمونه زبانی گرفته شد. در گفتار این کودکان خطای جبرانی وجود داشت. روش تحریک متمرکز به مادر آموزش داده شد و آن‌ها این روش را به مدت دو ماه با کودک خود در منزل تمرین می‌کردند. بعد از گذشت دو ماه از ارزیابی اول، از کودکان دوباره نمونه زبانی گرفته شد. درصد همخوان‌های صحیح (PCC)، درصد خطاهای جبرانی و خزانه همخوانی در نمونه زبانی هر کودک محاسبه گردید. 40 درصد از گفته‌ها توسط آسیب‌شناس گفتار و زبان دیگر گوش داده شد. پایایی میان ارزیاب در سطح بالایی بود. یافته‌ها: به دنبال مداخله، میزان درصد همخوان‌های صحیح تفاوت زیادی نکرد. کاهش درصد خطاهای جبرانی معنی‌دار نبود، اما خزانه همخوانی به طور معنی‌داری افزایش یافت. نتیجه‌گیری: در صورتی که این مطالعه با نمونه بیشتر و مدت زمان مداخله طولانی‌تر انجام شود، ممکن است تغییرات حاصل از آن بیشتر معنی‌دار باشد. با توجه به نتایجی که از این مطالعه به دست آمد، ممکن است بتوان از آن برای سایر کودکان دارای شکاف کام هم استفاده نمود و از تثبیت خطاهای آن‌ها در سیستم واج‌شناختی پیشگیری کرد و منجر به صرفه‌جویی در وقت و هزینه شد.

Title: بررسی و تعیین دیادوکوکینزیس دانش‌آموزان 7 تا 9 ساله طبیعی دبستان‌های شهر کرمانشاه

http://jrrs.mui.ac.ir/index.php/jrrs/article/view/688

مقدمه: تولید گفتار شامل مراحل طرح‌ریزی، برنامه‌ریزی و اجرا می‌باشد که هر کدام از این مراحل در مناطق خاصی از مغز صورت می گیرد. اختلال در هر کدام از این مراحل موجب اختلال در تولید گفتار و همچنین توانایی دیادوکوکینزیس می‌شود. تکالیف دیادوکوکینزیس در ارزیابی و درمان اختلالات گفتاری از جمله دیزآرتری و اختلالات مخچه کاربرد دارد. در این پژوهش سعی شد با بررسی دیادوکوکینزیس در کودکان طبیعی 7 تا 9 ساله معیاری از این جنبه در آن‌ها به دست آید. هدف این پژوهش، بررسی و تعیین دیادوکوکینزیس دانش‌آموزان 7 تا 9 ساله طبیعی دبستان‌های شهر کرمانشاه بود. مواد و روش‌ها: در مطالعه توصیفی- تحلیلی حاضر که به روش مقطعی انجام پذیرفت، 210 دانش‌آموز 7 تا 9 ساله (105 دختر و 105 پسر) در سه گروه سنی 7، 8 و 9 ساله (هر گروه شامل 70 نفر) دبستان‌های شهرستان کرمانشاه به طور تصادفی انتخاب شدند. دانش‌آموزان باید 20 بار تک هجای متشکل از همخوان و واکه /a/ و نیز واکه /u/ را جهت تعیین مدت زمان آن، تکرار می‌کردند. یافته‌ها: میانگین زمان تکرار تک هجایی‌ها در کودکان 7 ساله 05/5 ثانیه، در کودکان 8 ساله 68/4 ثانیه و در کودکان 9 ساله 47/4 ثانیه بود. نتیجه‌گیری: نتایج این مطالعه نشان داد که سن و نوع واکه گفتاری به کار گرفته شده می‌تواند روی توانایی دیادوکوکینزیس تاثیرگذار باشد که این یافته‌ها باید در تشخیص و درمان اختلالات تولیدی در نظر گرفته شود.

Title: بررسی عملکرد سیستم وابران در کودکان دارای اختلال واج‌شناختی 8-5 ساله

http://jrrs.mui.ac.ir/index.php/jrrs/article/view/530

مقدمه: بر خلاف سیستم آوران که اطلاعات زیادی از آن در دسترس است، اطلاعات محدودی در رابطه با سیستم وابران و نقش آن در پردازش جنبه‌های مختلف زبانی از جمله پردازش واج‌شناختی وجود دارد. هدف از این مطالعه، بررسی نقش سیستم وابران (Medial olivocochlear bundle یا MOCB) در کودکان دارای اختلالات واج‌شناختی بود. مواد و روش‌ها: 20 کودک 5 تا 8 ساله با شنوایی طبیعی در دو گروه قرار گرفتند. گروه شاهد شامل 11 کودک بدون اختلال واج‌شناختی وگروه مورد مطالعه شامل 9کودک دارای اختلال واج‌شناختی بودند. شرط ورود به مطالعه دارا بودن شنوایی در حد dBHL 15و یا بهتر، تمپانوگرام تایپ An و داشتن رفلکس در هر دو گروه بود. آزمون TEOAE و TEOAE Suppression به منظور بررسی سیستم وابران برای کلیه کودکان در دو گروه انجام گرفت. یافته‌ها: تفاوت قابل ملاحظه‌ای بین میانگین اثر مهار در آزمون TEOAE Suppression در دو گروه در فرکانس‌های مختلف وجود نداشت. نتیجه‌گیری: بر اساس نتایج به دست آمده در این بررسی، تغییری در MOCB کودکان دارای اختلالات واج‌شناختی مشاهده نگردید.

Title: مقایسه سازه‌های فرکانسی اول و دوم در کودکان دچار درجات مختلف افت شنوایی و کودکان دچار شنوایی عادی

http://jrrs.mui.ac.ir/index.php/jrrs/article/view/534

مقدمه: سازه‌های فرکانسی تحت تأثیر حرکات اندام‌های تولید و تغییرات شکل مجرای صوتی می‌باشند. در تولید واکه‌های افراد دچار افت شنوایی، حرکات زبان در بعد عمودی محدود و قوس زبان کاهش می‌یابد که منجر به مرکزی‌شدگی سازه‌های فرکانسی می‌شود. در مطالعه حاضر قصد بر این بود که با توجه به نبود اطلاعاتی جامع در گروه‌های مختلف دچار افت شنوایی و عدم مقایسه گروه‌های دچار افت شنوایی با یکدیگر در مطالعات بررسی شده، سازه‌های فرکانسی واکه‌های /u/، /i/ و /a/ در این کودکان و در مقایسه با کودکان هنجار بررسی شود. موادو روش‌ها: پژوهش توصیفی- تحلیلی حاضر بر روی 80 دانش‌آموز عادی و آسیب‌دیده شنوایی (متوسط، متوسط تا شدید، شدید و عمیق) انجام گرفت. از آزمودنی‌ها خواسته شد سه واکه /a/، /i/ و /u/ را سه بار بکشند. جهت ثبت سازه‌های فرکانسی، فایل‌های صوتی به نرم‌افزار Praat (نسخه 5.3.13) منتقل شد. برای هر متغیر، ابتدا آزمون ANOVA و سپس آزمون پست هاک Dunnett برای مقایسه گروه شاهد با گروه‌های دچار افت شنوایی و آزمون Duncan برای مقایسه بین گروه‌های دچار افت شنوایی انجام شد. یافته‌ها: در مقایسه گروه شاهد با سایر گروه‌های دچار افت شنوایی، 2F واکه‌های /a/ و /i/ بر خلاف 1F، بین گروه شاهد و تمام گروه‌های دچار افت شنوایی تفاوت معنی‌دار داشتند (a2F: گروه عادی- متوسط: 031/0 = P، گروه عادی- متوسط به شدید: 015/0 = P، گروه عادی- شدید: 023/0 = P، گروه عادی- عمیق: 0001/0 > P و i2F: گروه عادی- متوسط: 005/0 = P، گروه عادی- متوسط به شدید: 0001/0 > P، گروه عادی- شدید: 0001/0 > P، گروه عادی- عمیق: 0001/0 > P)، اما در مقایسه درون گروه‌های دچار افت شنوایی توسط آزمون Duncan مشخص شد که 1F واکه /u/ بیشترین تفاوت‌ها را بین گروه‌های دچار افت شنوایی نشان می‌دهد. نتیجه‌گیری: مقایسه گروه‌های مختلف نشان داد که 2F واکه‌های /a/ و /i/ تفاوت بیشتری نسبت به 2F واکه /u/ و همچنین 1F واکه‌های /a/، /u/ و /i/ در نشان دادن تفاوت گفتار طبیعی از غیر طبیعی دارند و بهتر می‌تواند تفاوت‌های واکه‌ای را نشان دهد. از این رو به نظر می‌رسد تفاوت بیشتر 2F واکه‌های/a/ و /i/ نسبت به سایر سازه‌ها در افراد دچار افت شنوایی نسبت به افراد دارای شنوایی هنجار بتواند دیدگاهی جدید در تشخیص و ارزیابی آسیب‌های واکه‌ای در این افراد ارایه دهد و به ارزیابی سریع‌تر و دقیق‌تر واکه‌ها در این کودکان منجر شود.

Title: بررسی ارتباط بین حداکثر مدت زمان آواسازی (MPT) با سن و سابقه تدریس در معلمان زن با و بدون شکایت صوتی در مدارس ابتدایی شهر تهران

http://jrrs.mui.ac.ir/index.php/jrrs/article/view/796

مقدمه: معلمان بزرگ‌ترین گروه کاربران حرفه‌ای صوت محسوب‌ می‌‌شوند که به واسطه شرایط شغلی خاص، در معرض اختلالات صوت بیشتری نسبت به دیگران قرار دارند. مطالعه حاضر با هدف، تعیین و مقایسه حداکثر مدت زمان آواسازی (Maximum phonation time یا MPT) و همچنین بررسی تأثیر سن و سابقه تدریس بر  MPTدر معلمان زن با و بدون شکایت صوتی در مدارس ابتدایی شهر تهران صورت گرفت. مواد و روش‌ها: روش انجام پژوهش حاضر توصیفی- تحلیلی، غیر مداخله‌ای و از نوع مقطعی- مقایسه‌ای بود که بر روی 80 معلم زن در دو گروه با و بدون شکایت صوتی انجام گرفت. اطلاعات زمینه‌ای از طریق مصاحبه استخراج شد و سپس برای ارزیابی MPT، از آزمودنی خواسته ‌‌شد با یک دم عمیق واکه /a/ را با حداکثر توان آواسازی به صورت کشیده تولید کند. این ارزیابی برای سه بار متوالی با فاصله‌ زمانی 1 دقیقه انجام گرفت و بیشترین زمان به عنوان حداکثر مدت زمان آواسازی در نظر گرفته شد. یافته‌ها:‌ میانگین MPT در گروه با شکایت صوتی (88/2 ± 88/12)، نسبت به‌ میانگین MPT در گروه بدون شکایت صوتی ‌(17/3 ± 97/15) تفاوت معنی‌داری داشت (05/0 > P). همچنین همبستگی معنی‌دار منفی بین سن و سابقه تدریس با MPT در دو گروه وجود داشت. نتیجه‌گیری: با بالا رفتن سن و سابقه تدریس،‌ میانگین MPT در هر دو گروه کاهش‌ می‌‌یابد و میانگین MPT در گروه با شکایت به صورت معنی‌داری پایین‌تر از گروه بدون شکایت است که می‌تواند نشان دهنده کیفیت صوت بدتر باشد.

Title: مروری بر آزمون‌های ارزیابی نگرش افراد مبتلا به لکنت

http://jrrs.mui.ac.ir/index.php/jrrs/article/view/615

مقدمه: لکنت اختلالی ارتباطی می‌باشد که شامل رفتارهای اصلی و ثانویه است. جهت ارزیابی، تشخیص و درمان مناسب لکنت نیاز است تا تمام رفتارهای این اختلال مورد توجه قرار گیرند. یکی از مشکلاتی که در زمینه تعمیم و تثبیت درمان افراد مبتلا به لکنت وجود دارد، نگرش‌های نا مناسبی است که به واسطه لکنت در این افراد به وجود آمده است. به همین دلیل به نظر می‌رسد شناسایی این نگرش‌ها و سعی بر تغییر آن‌ها نقش عمده‌ای در روند درمان لکنت دارد. هدف از مطالعه حاضر، معرفی اولیه آزمون‌های ارزیابی نگرش افراد مبتلا به لکنت بود. مواد و روش‌ها: جهت دسترسی کامل و جامع به ابزارهای بررسی نگرش افراد مبتلا به لکنت، جستجو در پایگاه‌های اطلاعاتی مختلف، کتاب‌ها و پایان‌نامه‌های مربوط به اختلال لکنت و همچنین سایت‌های اطلاع‌رسانی در این زمینه انجام پذیرفت. در مراحل بعدی و پس از بررسی آزمون‌ها، مواردی که جهت آشنایی با هر آزمون به آن‌ها نیاز بود، تعیین گردید. یافته‌ها: پس از بررسی نتایج، تعداد 22 آزمون در زمینه ارزیابی نگرش افراد مبتلا به لکنت یافت شد. تعداد 10 مورد از این آزمون‌ها مربوط به ارزیابی نگرش کودکان و بقیه موارد این آزمون‌ها مربوط به ارزیابی نگرش بزرگسالان مبتلا به لکنت بود. این آزمون‌ها در جداول جداگانه‌ای جهت آشنایی اولیه لیست‌بندی شدند. بعضی آزمون‌ها نیز دارای نکات قابل توجهی بودند که این موارد نیز توضیح داده شده‌اند. نتیجه‌گیری: با توجه به نتایج مقاله حاضر می‌توان بیان نمود که ارزیابی نگرش یکی از مراحل مهم در روند درمان لکنت می‌باشد که نیازمند توجه بیشتر آسیب‌شناسان گفتار و زبان به این مقوله است.
